# Antireflux Metal Stent for Initial Treatment of Malignant Distal Biliary Obstruction

**DOI:** 10.1155/2018/3805173

**Published:** 2018-01-31

**Authors:** Shinichi Morita, Yasuaki Arai, Shunsuke Sugawara, Miyuki Sone, Yasunari Sakamoto, Takuji Okusaka, Shigetaka Yoshinaga, Yutaka Saito, Shuji Terai

**Affiliations:** ^1^Department of Gastroenterology and Hepatology, Uonuma Institute of Community Medicine, Niigata University Hospital, 4132 Urasa, Minamiuonuma, Niigata 949-7302, Japan; ^2^Department of Diagnostic Radiology, National Cancer Center Hospital, 5-1-1 Tsukiji, Chuo-ku, Tokyo 104-0045, Japan; ^3^Department of Hepatology and Pancreatobiliary Internal Medicine, National Cancer Center Hospital, 5-1-1 Tsukiji, Chuo-ku, Tokyo 104-0045, Japan; ^4^Endoscopy Division, National Cancer Center Hospital, 5-1-1 Tsukiji, Chuo-ku, Tokyo 104-0045, Japan; ^5^Department of Gastroenterology and Hepatology, Niigata University Hospital, 754 Ichibancho, Asahimachidori, Chuo-ku, Niigata city, Niigata 951-8510, Japan

## Abstract

**Objectives:**

To compare the use of an antireflux metal stent (ARMS) with that of a conventional covered self-expandable metal stent (c-CSEMS) for initial stenting of malignant distal biliary obstruction (MDBO).

**Materials and Methods:**

We retrospectively investigated 59 consecutive patients with unresectable MDBO undergoing initial endoscopic biliary drainage. ARMS was used in 32 patients and c-CSEMS in 27. Technical success, functional success, complications, causes of recurrent biliary obstruction (RBO), time to RBO (TRBO), and reintervention were compared between the groups.

**Results:**

Stent placement was technically successful in all patients. There were no significant intergroup differences in functional success (ARMS [96.9%] versus c-CSEMS [96.2%]), complications (6.2 versus 7.4%), and RBO (48.4 versus 42.3%). Food impaction was significantly less frequent for ARMS than for c-CSEMS (*P* = 0.037), but TRBO did not differ significantly between the groups (log-rank test, *P* = 0.967). The median TRBO was 180.0 [interquartile range (IQR), 114.0–349.0] days for ARMS and 137.0 [IQR, 87.0–442.0] days for c-CSEMS. In both groups, reintervention for RBO was successfully completed in all patients thus treated.

**Conclusion:**

ARMS offers no advantage for initial stent placement, but food impaction is significantly prevented by the antireflux valve.

## 1. Introduction

For patients with unresectable malignant distal biliary obstruction (MDBO), endoscopic placement of a self-expandable metal stent (SEMS) is a widely accepted treatment for the relief of jaundice [1–4]. SEMS has been used not only as palliative therapy but also prior to chemotherapy. The survival time for patients with pancreatic cancer has been improving, due mainly in part to recent advances in chemotherapeutic intervention [5–7]. Therefore, prolongation of SEMS patency is desirable to allow continuation of anticancer treatment and thus improve patient prognosis.

SEMS remains patent for longer than a plastic stent because of its wide lumen [8, 9]. The diameter of the SEMS is generally more than 8 mm after placement, allowing a relative smoothness and reduction of sludge accumulation that can compromise stent function. However, SEMS dysfunction does occur occasionally after placement, leading to cholangitis that requires reintervention.

In cases of MDBO, the SEMS is usually placed across the papilla. However, this is reported to increase the risk of occlusion due to loss of sphincter function [10], and consequent duodenobiliary reflux can lead to sludge accumulation [11]. To prevent this and to prolong stent patency, the antireflux metal stent (ARMS) was developed, and many studies have reported that this is more effective than ordinary stents [12–18]. However, as reported by Ustundag et al., the superiority of the ARMS over the ordinary covered SEMS has not been verified, especially for initial stenting [19], and therefore the effectiveness of ARMS for MDBO remains controversial.

In the present study, we retrospectively compared the effectiveness of the ARMS versus the conventional covered SEMS (c-CSEMS) for initial stenting in patients with unresectable MDBO.

## 2. Materials and Methods

This study was approved by the institutional Human Investigation Committee of the National Cancer Center Japan (2014-322), and written informed consent was obtained from all patients in accordance with the tenets of the Declaration of Helsinki.

### 2.1. Patients

Between February 2013 and December 2014, 59 consecutive patients (31 men and 28 women; median age, 67 years; range, 23–88 years) with unresectable MDBO were treated with SEMS and enrolled in this study. Patients with hilar biliary obstruction and surgically altered gastrointestinal anatomy, or those with a performance status lower than 4 according to the Eastern Cooperative Oncology Group [20] scale, were excluded. The SEMS was placed endoscopically in all patients. c-CSEMS placement was performed in 27 consecutive patients (13 men and 14 women; median age, 65 years; range, 23–88 years; c-CSEMS group) between February 2013 and March 2014, and ARMS placement was performed in 32 consecutive patients (18 men and 14 women; median age, 71 years; range, 43–87 years; ARMS group) between March and December 2014. The underlying diseases were pancreatic cancer in 50 patients, bile duct cancer in 4, lymph node metastases in 4, and ampullary cancer in 1. The diagnosis was based on imaging modalities such as computed tomography, magnetic resonance imaging, and endoscopic retrograde cholangiopancreatography (ERCP). Malignancy was confirmed pathologically by endoscopic ultrasound-guided fine-needle aspiration, bile duct biopsy, or brushing cytology. All patients were followed up from the time of stent placement to the recurrent biliary obstruction (RBO) or death if the patients were not obstructed by their stents.

### 2.2. Stent Designs

The characteristics of the inserted stents are shown in Table S1. The ARMS [13] (Niti-S long covered ComVi stent, Taewoong Medical Inc., Gimpo, Korea) is manufactured on the basis of the Niti-S ComVi SEMS [21] and has a funnel-shaped antireflux valve attached at its distal end (Figure S1 A and B). The valve portion of the stent is 7 mm long. The inner and outer layers of this stent are braided with nitinol wire, and an e-polytetrafluoroethylene (e-PTFE) membrane is sandwiched between these layers. The axial force of the inner and outer layers is weak, and the membrane is not fixed to the wire mesh. Thus, the axial force of the ComVi stent is weak, and the membrane fully covers the stent. The ARMS employed was 10 mm in diameter, and the metallic portion was available in lengths of 60 mm and 80 mm.

The c-CSEMS used in this study was the Niti-S SUPREMO stent (Taewoong Medical Inc., Gimpo, Korea) [22], which is braided with nitinol and has characteristic irregularly knitted stent cells (Figure S1 C). The large and small cells alter the amount of radial force, which is able to increase and decrease to allow the stent to fit into the bile duct. The stent membrane is made of silicone integrated with wire mesh, fully covering the stent. It was 10 mm in diameter and was available in lengths of 60 mm and 80 mm. Both ends of this stent were slightly flared.

### 2.3. Procedures

All SEMSs were placed using an ERCP technique. All of the patients underwent ERCP with a standard side-viewing duodenoscope (JF260V or TJF260V; Olympus Optical Co., Tokyo, Japan). All procedures were performed under conscious sedation, and all patients received prophylactic antibiotics. Sphincterotomy was performed before stent insertion in all cases. After evaluation of biliary stricture by cholangiography, a 0.025-inch guidewire (Visiglide or Visiglide 2; Olympus Optical Co., Tokyo, Japan) was passed through the stricture and inserted into the hepatic bile duct. The c-CSEMS or ARMS delivery system was inserted into the bile duct over the prepositioned guidewire, and the stent was deployed under fluoroscopic and endoscopic guidance. In all cases, the stent was placed across the papilla and the metallic portion extended into the duodenum for approximately one centimeter. The length of the stent was determined on the basis of cholangiographic findings.

All patients were followed up clinically, and complete blood counts and liver function tests were performed within one week after stenting, being repeated every month thereafter.

If symptoms of RBO such as high fever, upper abdominal pain, and/or jaundice occurred, reintervention was performed if an endoscopic procedure was permissible. For cleaning of the lumina of problematic stents with modest sludge accumulation, a balloon catheter for stone removal was employed. On the other hand, stents that had become almost or completely occluded were grasped with alligator forceps and removed carefully. In cases where the stent had migrated proximally (i.e., into the common bile duct), the stent was similarly and carefully pulled up and out through the duodenum. A new metal stent was then deployed, and the replacement stent was the same type as the previous one.

### 2.4. Study Design

This study was designed as a single-institution retrospective study. Evaluation of stent treatment was based on the TOKYO criteria 2014, which is a standardized system for reporting the status of biliary stents [23]. The primary outcome of this study was the time to RBO (TRBO). RBO was defined as the recurrence of obstructive jaundice and/or cholangitis due to stent occlusion or migration. TRBO was defined as the length of time between stent placement and the point of RBO. The secondary outcomes included technical success, functional success, complications, causes of RBO, risk factors for RBO, and reintervention. Technical success was defined as successful deployment of a stent with sufficient coverage of the stricture. Functional success was defined as a 50% decrease in or normalization of the bilirubin level to a standard value used at our institution within 14 days of stent placement.

Causes of stent occlusion such as sludge accumulation and food impaction were determined when reintervention revealed a large amount of sludge or food residue, respectively, in an occluded SEMS. Stent migration was diagnosed when a reintervention revealed a completely or partially migrated SEMS as a cause of RBO.

### 2.5. Statistical Analysis

Statistical analyses were performed using SPSS software for Windows version 22.0 (SPSS Inc., Chicago, IL). Differences between groups were compared using the Mann–Whitney *U* test, and differences in proportions were compared using Fisher's exact test. Kaplan-Meier estimation of TRBO was performed, and survival curves were compared by the log-rank test. Patient death was treated as censored at the time of death. Univariate and multivariate analyses were performed to identify risk factors for RBO using the Cox proportional hazards model. The model included age (<70 versus ≧70 years), sex, stent type (ARMS versus c-CSEMS), tumor etiology, complicating cholangitis, tumor invasion of the duodenum, the level of serum total bilirubin (<3.0 versus ≧3.0 mg/dL), use of oral ursodeoxycholic acid after stenting, and adjuvant chemotherapy. Statistical significance was defined as *P* < 0.05. The statistical methods used in this study were reviewed by Stagen Co. Ltd.

## 3. Results

### 3.1. Patient Characteristics

Patient characteristics and clinical details of the ARMS and c-CSEMS groups are summarized in Table 1. There were no significant intergroup differences in sex, age, diagnosis, duodenal invasion, chemotherapy, or oral ursodeoxycholic acid use after stenting.

### 3.2. Outcomes

Technical success was achieved in all patients (100%). Functional success was achieved in 31 of the 32 patients (96.9%) in the ARMS group and 26 of the 27 patients (96.2%) in the c-CSEMS group. The median procedure time did not differ significantly between the groups.

Procedure-related complications occurred in two patients (6.2%) in the ARMS group and two (7.4%) in the c-CSEMS group; the intergroup difference was not significant. Moderate cholecystitis developed in one patient in the ARMS group and two in the c-CSEMS group. These patients underwent percutaneous transhepatic gallbladder drainage and were administered prophylactic antibiotics. Their condition improved in a short time without cholecystectomy. In the ARMS group, one patient suffered liver failure due to portal vein occlusion because of the pressure of stent expansion and the jaundice did not improve. There was no procedure-related mortality.

RBO occurred in 15 patients (48.4%) in the ARMS group and 12 (46.2%) in the c-CSEMS group. In the ARMS group, the causes of RBO were tumor overgrowth in 1 patient, sludge accumulation (Figure S2 A and B) in 9, and symptomatic migration in 5. Migration was distal in 3 patients and proximal in 2 (Figure S2 C). In the c-SEMS group, RBO was due to tumor overgrowth in 1 patient, sludge accumulation in 5, food impaction in 4, and distal migration in 2. The incidence of food impaction was significantly higher for c-CSEMS than for ARMS (*P* = 0.037). Outcomes of ARMS are shown in Table 2.

### 3.3. Time to Recurrent Biliary Obstruction

**Figure 1:**
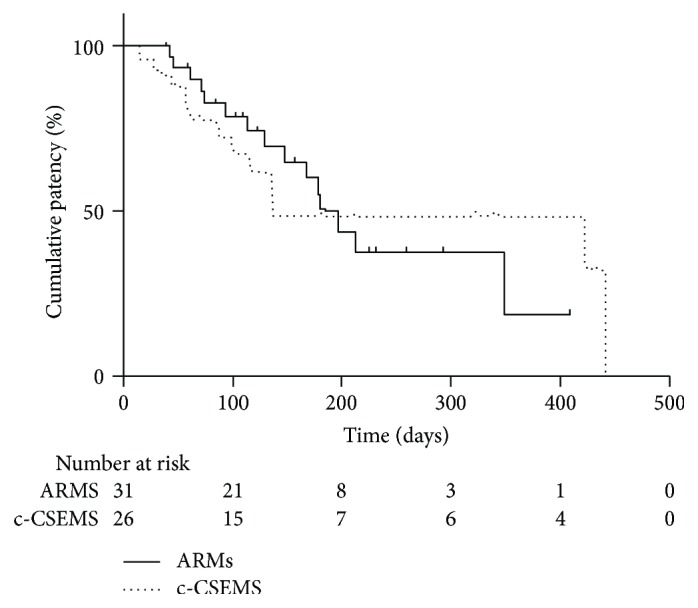
Kaplan-Meier curves showing TRBO of the ARMS and c-CSEMS groups (*P* = 0.967 by the log-rank test).

Kaplan-Meier estimation of TRBO was performed for patients achieving functional success. The results of the log-rank test showed no significant intergroup difference in TRBO (log-rank, *P* = 0.967; Figure 1). Rates of nonobstruction at 3, 6, and 12 months were 82%, 50%, and 19%, respectively, for ARMS and 72%, 48%, and 32%, respectively, for c-CSEMS. The median TRBO was 180.0 [interquartile range (IQR), 114.0 to 349.0] days for ARMS and 137.0 [IQR, 87.0 to 442.0] days for c-CSEMS.

### 3.4. Risk Factors for Recurrent Biliary Obstruction

The results of univariate and multivariate analyses for identifying RBO risk factors are shown in Table S2. There were no factors significantly predictive of RBO, including the stent type.

### 3.5. Reintervention

In the ARMS group, 14 patients underwent reintervention at the time of RBO. One patient improved after antibiotic administration alone. Two patients with modest sludge accumulation in the stent underwent cleaning of the stent lumen with a balloon catheter for stone removal and drainage with a nasal biliary drainage tube. Seven patients underwent removal of the occluded stent with alligator forceps and replacement with a new ARMS. Two patients with RBO due to proximal migration underwent careful removal of the stent from the common bile duct using alligator forceps without any problems and then received a replacement ARMS. Among 3 patients with RBO due to distal migration, 2 underwent removal of the migrated stent with a conventional endoscope, and in 1 patient, the stent had already been discharged externally when RBO occurred. All of these patients received a replacement ARMS.

In the c-CSEMS group, 10 patients underwent reintervention due to RBO. One patient improved after antibiotic administration alone. Four patients with modest sludge accumulation in the stent underwent cleaning of the stent lumen with a balloon catheter for stone removal and drainage with a nasal biliary drainage tube. In one patient, removal of the occluded stent was attempted with alligator forceps, but the stent could not be removed because of unusual resistance. In this patient, a new c-CSEMS was placed as a stent-in-stent deployment. Three patients underwent removal of the occluded stent with alligator forceps and received a new c-CSEMS. In two patients with RBO due to distal migration, the stents had already been discharged externally when RBO occurred. Both of these patients received a new c-CSEMS. In both groups, all reintervention procedures were performed endoscopically without any complications.

## 4. Discussion

Currently, SEMSs are used widely for unresectable MDBO, not only as palliative therapy [1–4] but also before chemotherapy [24, 25]. They are effective for improving the quality of life in patients with a poor prognosis. In patients with MDBO, the SEMS has been proven to retain its patency for longer than a plastic stent because of its wide caliber [8, 9] and therefore has become standard therapy. However, this approach is not ideal for biliary obstruction, as stent occlusion may occur for various reasons such as tumor growth, sludge accumulation, food impaction, and migration. Therefore, many attempts have been made to prolong stent patency [17, 26–29].

In most patients with MDBO, the SEMS needs to be placed through the papilla. However, this results in sphincter of Oddi dysfunction, a proven cause of RBO [10, 11, 30, 31], and may cause duodenobiliary reflux whereby intestinal fluid and food residue pass through in a retrograde direction, creating a biofilm on the SEMS and sludge build-up. Additionally, the wide lumen of the SEMS may allow direct clogging by food residues. In fact, Misra and Dwivedi [11] have reported that in all patients with MDBO and placement of a SEMS, barium reflux from the duodenum to the bile duct was evident. This has prompted the development of ARMS with an antireflux valve to prevent duodenobiliary reflux. Many valve shapes are available, and their effectiveness has been shown to be comparatively good (Table 3) [12–17, 32, 33]. Hamada et al. [13] have stated that subjects with metal stents that have become occluded due to duodenobiliary reflux are good candidates for ARMS. In fact, they reported good outcomes of ARMS as a reintervention for SEMS occlusion. Hu et al. [16] reported a randomized controlled trial involving 112 patients treated with a nipple-shaped partially covered ARMS versus uncovered SEMS as the first-line stent for MDBO. The ARMS retained its patency significantly longer, and the frequency of cholangitis after stenting was significantly lower than that for the uncovered SEMS. In that study, however, an uncovered SEMS was employed, and this is reported to have a shorter patency duration than a covered SEMS [26, 27]. Therefore, the basic SEMS structure might influence TRBO, and the true significance of the antireflux valve remains unclear. Lee et al. [32] developed a SEMS with a windsock-shaped antireflux valve, although its basic structure was that of a classical fully covered SEMS. They reported a statistically significant improvement of TRBO with ARMS. However, since the valve length of their study was 22 mm, there were concerns whether it could be deployed accurately via endoscopy.

In the present study, the valve of the ARMS was 7 mm long, and deployment was similar to that of the common SEMS. We deployed ARMS successfully in all patients initially treated for MDBO. However, as the TRBO in the ARMS group was not significantly different from that in the c-CSEMS group (180.0 versus 137.0 days, *P* = 0.967), we were unable to identify any benefit of ARMS. Univariate and multivariate analyses failed to reveal any significant risk factors for RBO, including the type of SEMS employed.

Although ARMS did prevent food impaction (*P* = 0.037), the rates of RBO due to sludge accumulation and stent migration were the same as those of c-CSEMS. There are several possible reasons for this. Although ARMS certainly prevents reflux of food residue, it cannot prevent reflux of fluids such as intestinal secretion from the duodenum and therefore formation of a biofilm on the inner membrane of the SEMS. Furthermore, formation of sludge in the valve tended to be more frequent, making the valve harder and stiffer over time, as well as shrinking the luminal space, thus leading to a sluggish and disturbed bile outflow. The antireflux valve designs with superior materials and structure have been expected, with the aim of preventing duodenobiliary reflux without interfering with antegrade bile flow. In addition, the stent migration rate in patients with RBO tended to be higher for ARMS than for c-CSEMS (16.1 versus 7.7%, *P* = 0.436). This might have been due to the nonflared stent edge of the ARMS, whereas the c-CSEMS was slightly flared at both ends. In fact, Hamada et al. [17] have reported that a newly designed ARMS with both ends flared may have a lower stent migration rate.

This is the first study to have demonstrated that, whereas the antireflux valve significantly reduces food impaction, the stent itself offers no significant advantage for initial stenting of MDBO.

Reintervention is a crucial factor affecting the prognosis of patients with MDBO. In particular, when an ARMS becomes dysfunctional, the antireflux valve has already accumulated much tough sludge. Therefore, cleaning of the stent lumen is insufficient for restoring patency, and replacement with a new stent is needed. Therefore, the possibility of SEMS removal is an important point that needs to be considered. In the present series, all patients requiring stent exchange underwent ARMS removal and replacement.

This study was limited in being retrospective and uncontrolled. Another limitation was the differences in stent structure and materials between the two groups. The basic structure of the ARMS is similar to that of the Niti-S ComVi stent [21]. The type of knitting of the ComVi stent also differs from that of the Niti-S SUPREMO stent [22], corresponding to the c-CSEMS we employed. Moreover, the axial force of the ComVi stent is lower than that of the SUPREMO stent. Stents with low axial force tend to show a longer TRBO [34], and therefore the difference in axial force produced by the basic SEMS structure cannot be ignored. Furthermore, the materials of the inner luminal surfaces differed, being a silicone in the c-CSEMS and an e-PTPF membrane in the ARMS. The silicone membrane is smoother than e-PTPF membrane. These differences in the inner membrane may affect the frequency of sludge accumulation or the degree of duodenobiliary reflux. Therefore, randomized controlled trials comparing ARMS with c-CSEMS with the same underlying structure and materials, except for the antireflux valve, will be needed to determine the most suitable stent for initial treatment of MDBO.

## 5. Conclusions

The present study was unable to confirm the superiority of ARMS for initial treatment of MDBO. However, RBO due to food impaction was significantly prevented by the antireflux valve. There is a possibility that the SEMS structure could be further improved or more cases suitable for use of the antireflux system identified. Further investigations are needed to evaluate the superiority of ARMS for initial treatment of MDBO.

## Figures and Tables

**Table 1:** Patients' characteristics and clinical details of the ARMS and c-CSEMS groups.

	ARMS group	c-CSEMS group	*P* value
Number of patients	32	27	
Gender (male/female)	18/14	13/14	0.606
Median age (range) (years)	71 (43–87)	65 (23–88)	0.825
Diagnosis			0.901
Pancreatic cancer	28	22	
Bile duct cancer	3	1	
Ampullary cancer	0	1	
Lymph node metastases	1	3	
Duodenal invasion (%)	7 (21.9)	9 (33.3)	0.386
Chemotherapy (%)	22 (68.8)	17 (63.0)	0.640
Oral ursodeoxycholic acid (%)	7 (21.9)	9 (33.3)	0.386

ARMS: antireflux metal stent; c-CSEMS: conventional covered self-expandable metal stent.

**Table 2:** Clinical outcomes of the ARMS and c-CSEMS groups.

	ARMS group (*n* = 32)	c-CSEMS group (*n* = 27)	*P* value
Technical success (%)	32/32 (100)	27/27 (100)	—
Functional success (%)	31/32 (96.9)	26/27 (96.2)	1.000
Median procedure time (min)	30 (15–50)	40 (20–70)	0.735
Complications (%)	2 (6.2)	2 (7.4)	1.000
Cholecystitis	1	2	0.588
Liver failure	1	0	1.000
Recurrent biliary obstruction (%)	15/31 (48.4)	12/26 (46.2)	0.866
Overgrowth	1	1	1.000
Sludge accumulation	9	5	0.584
Food impaction	0	4	0.037^∗^
Migration	5	2	0.436
	Distal 3	Distal 2	
	Proximal 2	Proximal 0	
Reintervention (%)	14/15 (93.3)	11/12 (91.7)	1.000
Cleaning and drainage	2	4	
Additional stenting (stent in stent)	0	1	
Replacing stent	12	6	

^∗^ *P* < 0.05. ARMS: antireflux metal stent; c-CSEMS: conventional covered self-expandable metal stent.

**Table 3:** Review of published articles on ARMS.

	Study design	No. of patients	Type of SEMS	Antireflux valve	TRBO	Complications	Cause of stent obstruction					
							Sludge	Food impaction	Ingrowth	Overgrowth	Migration	Others
Hu et al. (2011) [12]	Prospective, single arm	23	UC 3, PC 20	Cross shaped	14 mo	Bleeding 1	0	0	1	2	3	
Lee et al. (2013) [15]	Prospective, single arm	32	UC	S-type	14.4 mo	None	6	0	4	0	1	
Kim et al. (2013) [14]	Prospective, single arm	5	PC	Wineglass shaped	64 d (4–235)	None	4	0	0	0	0	
Hamada et al. (2014) [13]	Prospective, single arm	13	FC	Funnel shaped	Not reached	None	1	0	0	0	4	Unknown 1
Hu et al. (2014) [16]	RCT	ARMS 56	PC	Nipple shaped	13.0 ± 3.4 mo	Pancreatitis 1, cholangitis 1, cholecystitis 1, bleeding 1	2	0	3	1	5	Unknown 6
		SEMS 56	UC	—	10 ± 1.2 mo	Pancreatitis 2, cholecystitis 1, bleeding 1	2	0	14	5	1	Unknown 7
Hamada et al. (2015) [17]	Prospective, single arm	8	FC	Funnel shaped	71 d	Cholecystitis 1	2	0	0	0	1	Hemobilia 1
Lee et al. (2016) [32]	RCT	ARMS 39	PC	Windsock shaped	407 d	Pancreatitis 2, cholangitis 1	4	—	1	0	3	Valve dysfunction 2, hemobilia 1
		SEMS 38	PC	—	220 d	Pancreatitis 4, cholangitis 2	8	—	3	2	3	
Hamada et al. (2016) [33]	Retrospective, single arm	20	FC	Funnel shaped	246 d	Pancreatitis 3, cholecystitis 1, liver abscess 1	3	2	0	0	2	
Present study	Retrospective	ARMS 32	FC	Funnel shaped	180 d	Cholecystitis 1, liver failure 1	8	0	0	1	5	
		SEMS 27	FC	—	137 d	Cholecystitis 2	3	4	0	1	3	

TRBO: time to recurrent biliary obstruction; ARMS: antireflux metal stent; RCT: randomized controlled trial; SEMS: self-expandable metal stent; UC: uncovered; PC: partially covered; FC: fully covered.

## Supplementary Materials

Supplementary Materials Supplementary Tables. Table S1: features of the antireflux metal stent (ARMS) and conventional covered self-expandable metal stent (c-CSEMS). Table S2: the results of univariate and multivariate analyses for identifying RBO risk factors. Supplementary Figures. Figure S1: (A) antireflux covered metal stent (Niti-S long covered ComVi stent, Taewoong Medical Inc.). A funnel-shaped antireflux valve was attached at the distal end; (B) endoscopic view showing ARMS placed with its distal end in the duodenal lumen; (C) conventional covered metal stent (Niti-S SUPREMO, Taewoong Medical Inc.). The knitted structure includes large and small cells. Figure S2: (A) an ARMS was occluded because of substantial sludge sticking to its valve; (B) a vertical section of the occluded ARMS. A portion of the antireflux valve was filled with sludge (white arrowheads); (C) an ARMS (white arrows) migrated into the common bile duct (proximal migration).
