# Supplementary material for: Antireflux Metal Stent for Initial Treatment of Malignant Distal Biliary Obstruction
Source: Gastroenterol Res Pract. 2018 Jan 31;2018:3805173. doi: 10.1155/2018/3805173 (PMC5831319; doi:10.1155/2018/3805173)
Supplement: Supplementary Materials — Supplementary Tables. Table S1: features of the antireflux metal stent (ARMS) and conventional covered self-expandable metal stent (c-CSEMS). Table S2: the results of univariate and multivariate analyses for identifying RBO risk factors. Supplementary Figures. Figure S1: (A) antireflux covered metal stent (Niti-S long covered ComVi stent, Taewoong Medical Inc.). A funnel-shaped antireflux valve was attached at the distal end; (B) endoscopic view showing ARMS placed with its distal end in the duodenal lumen; (C) conventional covered metal stent (Niti-S SUPREMO, Taewoong Medical Inc.). The knitted structure includes large and small cells. Figure S2: (A) an ARMS was occluded because of substantial sludge sticking to its valve; (B) a vertical section of the occluded ARMS. A portion of the antireflux valve was filled with sludge (white arrowheads); (C) an ARMS (white arrows) migrated into the common bile duct (proximal migration). [file 3805173.f1.docx]

**Supplementary Material**

**Antireflux metal stent for initial treatment of malignant distal biliary obstruction**

Shinichi Morita,* Yasuaki Arai, Shunsuke Sugawara, Miyuki Sone, Yasunari Sakamoto, Takuji Okusaka, Shigetaka Yoshinaga, Yutaka Saito, Shuji Terai

***Corresponding author:** Shinichi Morita, MD, PhD, Assistant Professor, Department of Gastroenterology and Hepatology, Uonuma Institute of Community Medicine, Niigata University Hospital, 4132 Urasa, Minamiuonuma City, Niigata, 949-7302, Japan. Telephone: +81-25-777-3200; Fax: +81-25-777-2811; Email: m0riz0u@extra.ocn.ne.jp

**Supplementary Tables**

**Table S1:** Features of the antireflux metal stent (ARMS) and conventional covered self-expandable metal stent (c-CSEMS).

|  | **Niti-S long-covered ComVi stent**  **(ARMS)** | **Niti-S SUPREMO stent**  **(c-CSEMS)** |
| --- | --- | --- |
| **Design** | Braided,  Fully covered with a funnel shaped antireflux valve | Braided,  Fully covered |
| **Wire** | Nitinol | Nitinol |
| **Knitting** | Hooking uniform stent cells | Large and small sizes of stent cells |
| **Cover membrane** | e-PTFE | Silicon |
| **Structure** | Sandwich structure with membrane and two meshes | Integration of the membrane and mesh |
| **Stent edge** | Non-flared,  Antireflux valve at distal end | Slightly flared at both ends |
| **Radial force　［N］^a^** | 7.67 | 6.43 |
| **Axial force　［N］^a^** | 0.04 | 0.56 |

^a^ The data of radial and axial forces of ARMS are reference values from the data of Niti-S

ARMS, Antireflux metal stent; c-CSEMS, Conventional covered self-expandable metal stent; e-PTFE, e-polytetrafluoroethylene

ComVi stent. Data cited from Refs. [21] and [22].

**Table S2:** The results of univariate and multivariate analyses for identifying RBO risk factors.

|  | **Univariate analysis** | | **Multivariate analysis** | |
| --- | --- | --- | --- | --- |
|  | OR (95%CI) | *p* value | OR (95%CI) | *p* value |
| Sex, male | 0.63　(0.29-1.40) | 0.259 | 0.76 (0.32-1.80) | 0.528 |
| Age > 70 | 1.95 (0.86-4.42) | 0.108 | 2.00 (0.85-4.72) | 0.115 |
| Pancreatic cancer | 0.98 (0.34-2.86) | 0.971 |  |  |
| Complicated cholangitis | 1.18 (0.2-2.68) | 0.692 |  |  |
| Duodenal invasion | 0.85 (0.32-2.28) | 0.746 | 1.03 (0.35-3.01) | 0.958 |
| ARMS | 1.02 (0.46-2.28) | 0.967 | 0.92 (0.40-2.10) | 0.834 |
| Chemotherapy | 1.74 (0.52-5.88) | 0.692 | 2.00 (0.58-6.88) | 0.274 |
| UDCA | 1.15 (0.47-2.79) | 0.761 |  |  |
| T.Bil ≧3.0 mg/dL | 0.84 (0.38-1.86) | 0.664 |  |  |

ARMS, Antireflux metal stent; UDCA, Ursodeoxycholic acid; T.Bil, Serum total bilirubin level; OR, Odds ratio; CI, Confidence interval.

**Supplementary Figures**


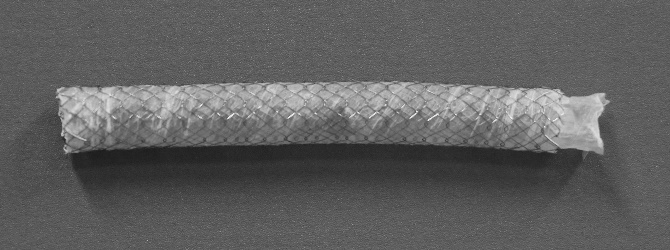

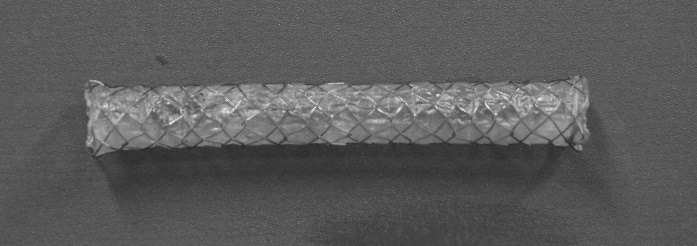


**B**

**A**


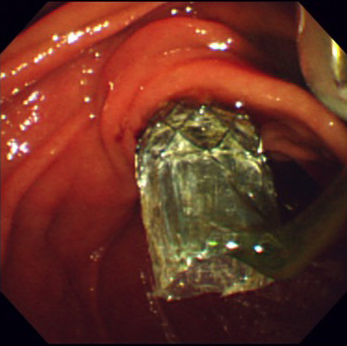


**C**

**Figure S1:** (A) Antireflux covered metal stent (Niti-S long covered ComVi stent, Taewoong Medical Inc.). A funnel-shaped antireflux valve was attached at the distal end; (B) Endoscopic view showing ARMS placed with its distal end in the duodenal lumen; (C) Conventional covered metal stent (Niti-S SUPREMO, Taewoong Medical Inc.). The knitted structure includes large and small cells.


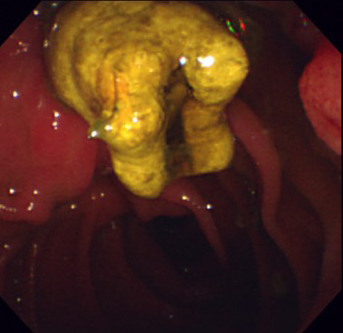

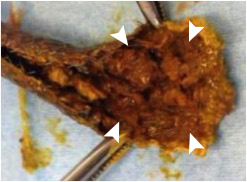


**B**

**A**


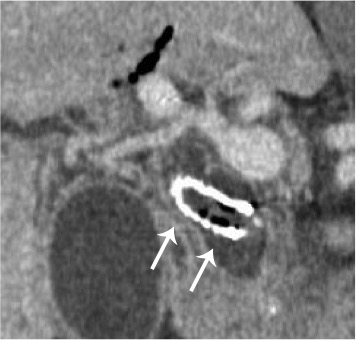


**C**

**Figure S2:** (A) An ARMS was occluded because of substantial sludge sticking to its valve; (B) A vertical section of the occluded ARMS. A portion of the antireflux valve was filled with sludge (white arrowheads); (C) An ARMS (white arrows) migrated into the common bile duct (proximal migration)
